# Inflammation, atrial fibrillation, and the potential role for colchicine therapy

**DOI:** 10.1016/j.hroo.2021.03.011

**Published:** 2021-04-02

**Authors:** Bibin Varghese, David I. Feldman, Christopher Chew, Eva Valilis, Roger S. Blumenthal, Garima Sharma, Hugh Calkins

**Affiliations:** ∗ Ciccarone Center for the Prevention of Cardiovascular Disease, Division of Cardiology, Johns Hopkins University School of Medicine, Baltimore, Maryland; † University of North Carolina School of Medicine, Chapel Hill, North Carolina; ‡ Department of Medicine, Division of Cardiology, Johns Hopkins Hospital, Baltimore, Maryland

**Keywords:** Atrial fibrillation, Catheter ablation, Colchicine, Electrophysiology, Inflammation, Prevention

## Abstract

Increasing evidence suggests that the “NACHT-LRR and PYD domain-containing protein 3” (NLRP3) inflammasome plays an important role in atherosclerotic cardiovascular disease (ASCVD). Recent preclinical evidence has suggested that the NLRP3 inflammasome may play a prominent role in the pathogenesis of atrial fibrillation (AF). As such, the therapies that have shown efficacy in reducing ASCVD events may also prove beneficial in AF. In this article, we review the findings that implicate the NLRP3 inflammasome in the pathogenesis of AF, discuss existing evidence behind the use of anti-inflammatory agents for AF, and discuss the future role that colchicine and other anti-inflammatory agents may play in the prevention and treatment of AF.

**Key Findings**

▪ The NLRP3 inflammasome may play a prominent role in the pathogenesis of atrial fibrillation (AF).
▪ Anti-inflammatory therapies such as colchicine have shown benefits in reducing recurrence of AF after surgery or ablation.
▪ Future randomized controlled trials are necessary to determine the efficacy of colchicine therapy for primary prevention and treatment of AF.

## Introduction

Atrial fibrillation (AF), the most common arrhythmia worldwide, affects 2%–3% of the population and is associated with significant morbidity and mortality.^1^^,^^2^ AF is commonly preceded by structural remodeling of the atrial myocardium, which predisposes to impaired electrical conduction.^3^ The genesis of AF begins with a focal trigger of ectopic firing that initiates a reentrant wave in a vulnerable atrial substrate. The onset of AF leads to further atrial remodeling, which facilitates maintenance and progression of AF from paroxysmal AF (pAF) to persistent and longstanding persistent AF.^4^, ^5^, ^6^, ^7^

Many nonmodifiable and modifiable risk factors, such as age, sex, ion channel mutations, hypertension, diabetes, obesity, and obstructive sleep apnea, have been linked to the progression of AF.^3^ However, whether there is a common fundamental mechanism leading to clinical AF remains unknown.^8^ Enhanced inflammatory signaling has been previously proposed as one potential link in the pathogenesis of AF.^9^ The association between inflammation and AF was first noted over 20 years ago with the high incidence rate of postoperative AF (up to 50%) after cardiac surgery.^10^ In studies of postoperative AF, elevated inflammatory markers such as interleukin-1β (IL-1β), IL-6, and C-reactive protein (CRP) temporally correlated with onset of AF. In addition, randomized controlled trials (RCTs) demonstrated that prophylactic anti-inflammatory therapies after cardiac surgery also reduced the risk of postoperative AF.^11^ Additional prospective cohort studies also suggested that higher CRP levels predicted onset of nonsurgical AF and lone AF in the general population.^9^ However, Mendelian randomization studies of patients with increased CRP levels as a result of genetic polymorphisms did not increase the incidence of AF, suggesting that CRP levels were a marker of increased risk but did not play a pathophysiological role in the genesis of AF.^12^ As such, it remained unclear if inflammatory processes precipitated AF or were simply a marker of increased risk.

A recent study by Yao and colleagues^13^ suggests that the “NACHT-LRR and PYD domain-containing protein 3” (NLRP3) inflammasome may play a causal role in the pathogenesis of AF. In this article, we review the findings from this study, discuss the evidence behind the use of anti-inflammatory agents for AF, and discuss the future role that colchicine may play in the prevention and treatment of AF.

**Figure 1:**
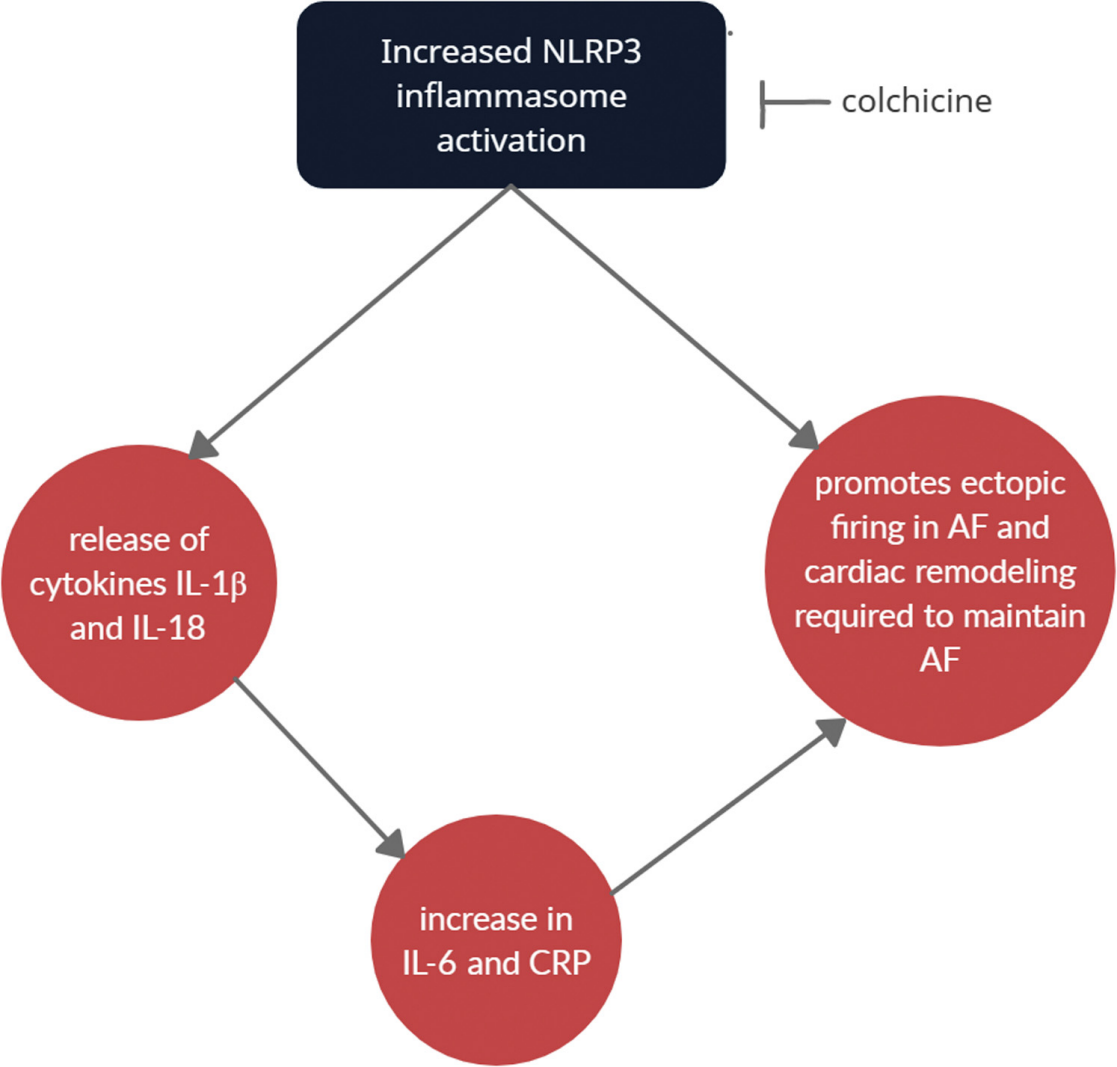
NLRP3 activation leads to downstream electrical remodeling within cardiomyocytes that result in increased ectopic firing and shortened atrial refractory period, thereby creating a reentry substrate. In addition, it leads to downstream cytokine release that promotes atrial remodeling necessary to maintain atrial fibrillation.

Inflammasomes are intracellular protein complexes that are responsible for activation of inflammatory signaling.^14^ They form in response to cellular detection of a broad range of signals including microbial motifs, danger signals, environmental irritants, etc, and serve as a first line of defense against pathogens.^4^ NLRP3 is a type of inflammasome that has been noted in numerous cell types including immune cells, epithelial cells, and cardiomyocytes. Activation of the NLRP3 inflammasome causes the release of cytokines such as IL-1β and IL-18, which leads to a cascade of downstream inflammatory signaling, resulting in the increase of IL-6. An increase in high-sensitivity C-reactive protein (hs-CRP), a biomarker of inflammation, has also been shown to occur with inflammasome activation. IL-6 and hs-CRP have been shown to independently predict future cardiovascular events^4^^,^^15^ (Figure 1).

The study by Yao and colleagues demonstrated that NLRP3 inflammasomes are enhanced in atrial cardiomyocytes of experimental models of AF, which promotes both the ectopic firing necessary to initiate AF and the remodeling of substrate needed to maintain and promote AF.^4^^,^^13^ In mouse models with constitutively active NLRP3 within cardiomyocytes, NLRP3 activation resulted in increased ectopic firing as a result of enhanced sarcoplasmic reticulum calcium release. It also led to electrical remodeling via transcriptional modifications that promoted a shortened atrial effective refractory period, thereby creating a reentry substrate. In addition, the enhanced inflammatory signaling resulted in activation of inflammatory cells that promote atrial fibrosis, thereby leading to the development of AF-maintaining substrate. The use of novel NLRP3 inhibitors or ablation of NLRP3 in the mouse models prevented the development of AF, supporting a causal link between NLRP3 activation within cardiomyocytes and AF. This study is consistent with prior results that have shown that inflammatory markers such as IL-6 and IL-1β, which are downstream signaling cytokines of NLRP3 inflammasome activation, correlate with progression of AF and also freedom from recurrence after AF ablation^4^ (Figure 1).

Recent clinical trials have demonstrated the importance of targeting the NLRP3 inflammasome pathway in reducing future atherosclerotic cardiovascular disease (ASCVD) events. The Canakinumab Anti-Inflammatory Thrombosis Outcomes Study (CANTOS) demonstrated that canakinumab, a monoclonal antibody against IL-1β, when given to patients with elevated hsCRP (≥2 mg/L) following a myocardial infarction, resulted in a 7%–15% reduction of cardiovascular events.^16^ However, the high cost and adverse side effect profile of canakinumab resulted in exploration of alternative anti-inflammatory agents such as colchicine.

Colchicine is an inexpensive anti-inflammatory medication that is commonly used for the treatment of gout and pericarditis.^17^ Colchicine works by inhibiting tubulin polymerization and has also been shown to interfere with the NLRP3/ IL-1β signaling pathway.^17^ The 2013 Low Dose Colchicine (LoDoCo) trial, the Colchicine Cardiovascular Outcomes (COLCOT) trial, and the LoDoCo 2 trial have demonstrated that treatment with colchicine for secondary prevention resulted in a reduction in future ASCVD events.^17^, ^18^, ^19^ Given the role of the NLRP3 inflammasome in both ASCVD and AF, we review the current evidence evaluating the role of anti-inflammatory therapies for treatment and prevention of AF.

## Anti-inflammatory therapies in AF

**Figure 2:**
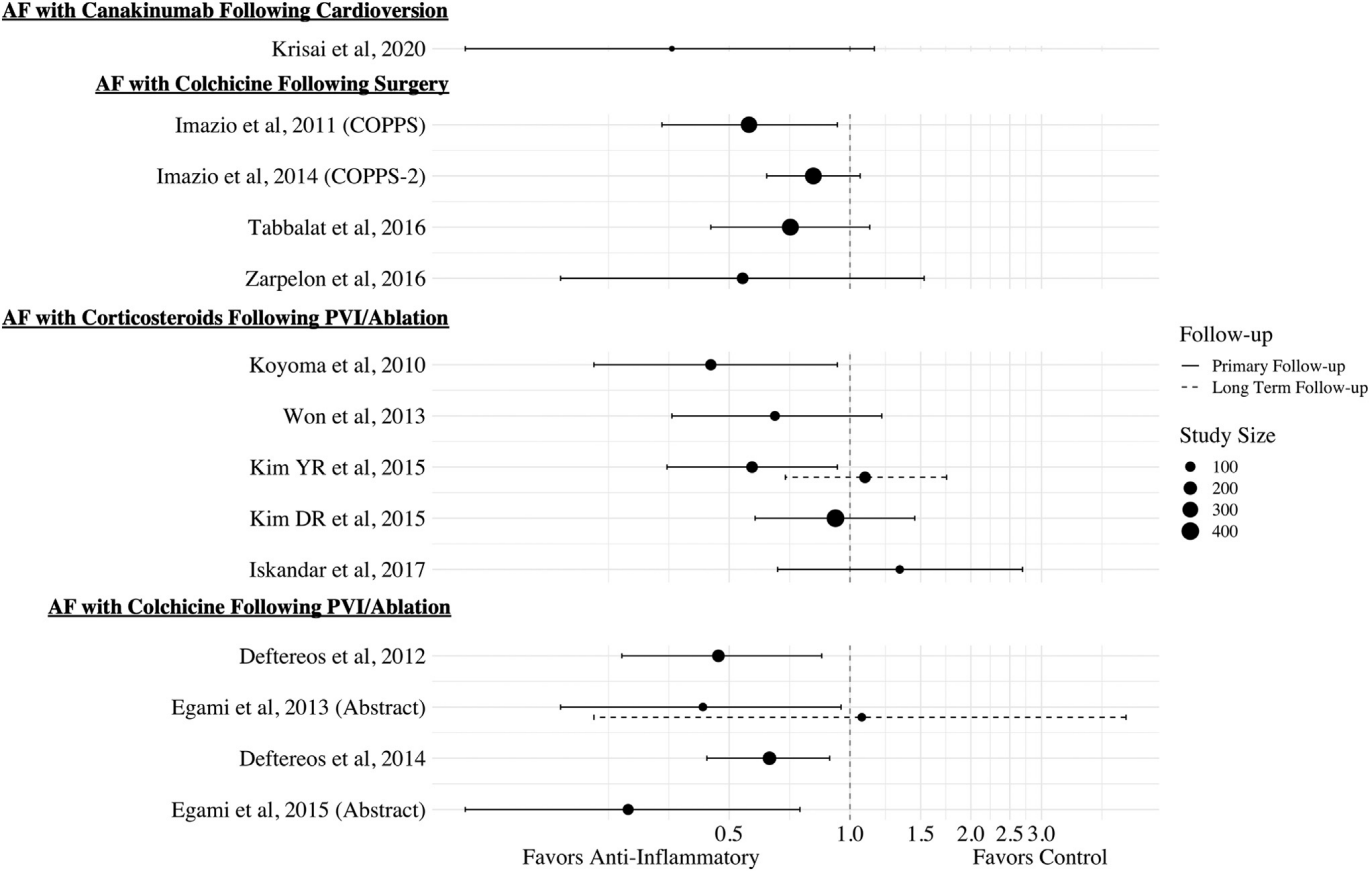
There appears to be a trend towards benefit of steroid therapy to treat atrial fibrillation (AF) after cardiac surgery. Although the results for steroid treatment after AF ablation to prevent recurrence appear to be equivocal, there is a clearer trend toward benefit with colchicine therapy. Further study is required to evaluate the benefit of canakinumab after cardioversion.

Currently, therapeutic strategies seek to minimize symptoms and avoid the complications associated with AF. Even procedural options such as ablation and cardioversion are complicated by high recurrence rates.^3^^,^^4^ However, current approaches do not target the potential key inflammatory mediators in the pathogenesis of AF. Although anti-inflammatory therapies such as canakinumab, colchicine, and steroids have shown ability to attenuate NLRP3-mediated inflammation, studies evaluating their role in the treatment and prevention of AF are limited^20^ (Table 1, Figure 2).

**Table 1:** Outcomes of atrial fibrillation recurrence with anti-inflammatory therapies

Study	Study type	Sample size	Intervention	Primary endpoint	Median follow-up	Effect estimate (95% CI)	Deduction
AF with canakinumab following cardioversion							
Krisai et al, 2020	Randomized controlled trial	24	Canakinumab	AF recurrence at 6 months	6 months	HR: 0.36 (0.11; 1.15)	Neutral
AF with colchicine following surgery							
Imazio et al, 2011 (COPPS)	Randomized controlled trial	336	Colchicine	Post-op AF at 1 month	1 month	RR: 0.56 (0.34; 0.93)	Positive
Imazio et al, 2014 (COPPS-2)	Randomized controlled trial	360	Colchicine	Post-op AF within 3 months	3 months	RR: 0.81 (0.62; 1.06)	Neutral
Tabbalat et al, 2016	Randomized controlled trial	360	Colchicine	Post-op AF after 1 week	8 days	RR: 0.71 (0.45; 1.12)	Neutral
Zarpelon et al, 2016	Randomized controlled trial	140	Colchicine	Post-op AF after 2 weeks	14 days	RR: 0.54 (0.19; 1.53)	Neutral
AF with corticosteroids following PVI/ablation							
Koyoma et al, 2010	Randomized controlled trial	125	Hydrocortisone and prednisolone	AF recurrence at 14 months	14 months	HR: 0.45 (0.23; 0.93)	Positive
Won et al, 2013	Prospective cohort study	89	Low-dose hydrocortisone	AF recurrence at 12 months	12 months	RR: 0.65 (0.36; 1.20)	Neutral
Kim YR et al, 2015	Randomized controlled trial	138	Methylprednisolone	AF recurrence at 3 months AF recurrence at 24 months	3 months 24 months	RR: 0.57 (0.35; 0.93) RR: 1.09 (0.69; 1.74)	Positive Neutral
Kim DR et al, 2015	Randomized controlled trial	407	Hydrocortisone and methylprednisolone	AF recurrence at 12 months	12 months	RR: 0.92 (0.58; 1.45)	Neutral
Iskandar et al, 2017	Randomized controlled trial	60	Prednisone	AF recurrence at 12 months	12 months	RR: 1.33 (0.66; 2.69)	Neutral
AF with colchicine following PVI/ablation							
Deftereos et al, 2012	Randomized controlled trial	170	Colchicine	AF recurrence at 3 months	3 months	RR: 0.47 (0.27; 0.85)	Positive
Egami et al, 2013 (Abstract)	Randomized controlled trial	62	Colchicine	AF recurrence at 2 weeks AF recurrence at 2 months	2 weeks 2 months	RR: 0.43 (0.19; 0.95) RR: 1.07 (0.23; 4.87)	Positive Neutral
Deftereos et al, 2014	Randomized controlled trial	206	Colchicine	AF recurrence at 12 months	12 months	RR: 0.63 (0.44; 0.89)	Positive
Egami et al, 2015 (Abstract)	Prospective cohort study	122	Colchicine	AF recurrence at 12 months	12 months	RR: 0.28 (0.11; 0.75)	Positive

AF = atrial fibrillation; CI = confidence interval; HR = hazard ratio; Post-op = postoperative; PVI = pulmonary vein isolation; RR = relative risk.

Krisai and colleagues^21^ evaluated the use of canakinumab after electrical cardioversion in 24 patients with persistent AF. After achieving sinus rhythm, patients treated with canakinumab vs placebo were followed to determine recurrence rate of AF after 6 months. Although there was a lower incidence of AF recurrence, the results were not statistically significant; the small sample size, however, was a significant limitation.^21^

Several RCTs have evaluated the role of colchicine for prevention of post–cardiothoracic surgery AF.^10^ The Colchicine for the Prevention of the Postcardiotomy Syndrome (COPPS) Atrial Fibrillation Substudy showed a reduced incidence of pAF in patients treated with colchicine for 1 month after pericardiotomy. A follow-up trial (COPPS-2) was conducted to evaluate patients undergoing any cardiac surgery except cardiac transplantation. Although the on-treatment subanalysis revealed a beneficial effect of colchicine for AF, this was not statistically significant based on an intention-to-treat analysis. Other studies have noted no benefit with colchicine in post-revascularization surgeries or in other open heart surgery patients.^22^^,^^23^

Patients who undergo AF ablation are known to have high levels of endocardial inflammation, including elevated IL-6 and hsCRP, in the first 3 months following the procedure, which may be associated with the 40%–50% rate of early and late AF recurrence.^24^^,^^25^ As such, several studies have evaluated the role of anti-inflammatory therapies such as colchicine or steroids after pulmonary vein isolation (PVI) and AF ablation to prevent recurrence of AF.^10^^,^^25^

The results with steroids have been inconsistent. In a small RCT by Koyama and colleagues^26^ including 125 patients undergoing PVI and ablation, the use of hydrocortisone on the day of AF ablation and prednisolone for 3 days after resulted in the decrease in immediate and late recurrence of AF. However, a similar study by Kim and colleagues^27^ using methylprednisolone showed there was a decrease in early recurrence rates, but no difference in late recurrence rates at the 24-month follow-up.

In the prospective, randomized, double-blinded Steroid-AF Study, 60 patients who failed antiarrhythmic therapy were randomized to prednisone or placebo after ablation for pAF.^28^ Prednisone did not reduce early or late recurrence of AF, and in fact, there was a trend towards higher rates of early AF recurrence with prednisone use. Two studies that evaluated the efficacy of a single-dose steroid injection after AF ablations did not show any benefit in preventing AF recurrence.^29^^,^^30^

In 2012, Deftereos and colleagues^31^ conducted the first study evaluating the efficacy of colchicine therapy after PVI and AF ablation in preventing AF recurrence. In this double-blinded placebo-controlled RCT, 161 patients undergoing ablation for pAF were treated with colchicine for 3 months after the procedure. Treatment with colchicine significantly reduced incidence of AF recurrence (16% vs 33.5%, *P* = .01).^31^

A follow up study by Deftereos and colleagues^32^ including patients who underwent ablation and treatment with colchicine for 3 months showed similar long-term reduction in AF recurrence rates at the 15-month follow-up (31% vs 50%, *P* = .01). In a 2013 prospective study, colchicine was effective at preventing AF recurrences within 2 weeks after ablation, but the effect was no longer present between 2 weeks and 2 months postablation.^33^ Several combined meta-analyses including postsurgical and postablation patients have evaluated the role of colchicine for preventing recurrence of AF after ablation, with 5 out of 6 meta-analyses suggesting a protective role of colchicine, whereas only 1 study was neutral.^34^, ^35^, ^36^, ^37^, ^38^, ^39^

To better understand the variable effect of colchicine for AF recurrence prevention, 122 patients with preprocedure computed tomography imaging undergoing AF ablation and treatment with colchicine were evaluated.^40^ The evaluation identified that patients with larger left atrial epicardial adipose tissue (LA-EAT) volumes were more likely to benefit from colchicine therapy than those who had smaller LA-EAT.^40^, ^41^, ^42^ In addition, LA-EAT has been linked to LA arrhythmogenicity and may be helpful in distinguishing who may benefit from anti-inflammatory therapy after AF ablation.^41^^,^^42^

## Limitations

In the studies evaluating the use of anti-inflammatory therapies for postprocedure AF, the role of the NLRP3 inflammasome pathway remains unclear. Although the inflammatory markers that are elevated in postprocedure AF (IL-1β, IL-6, and CRP) are similar to those after NLRP3 inflammasome activation, future studies that demonstrate the role of the NLRP3 inflammasome in postprocedure AF are necessary. In addition, studies that evaluate the role of the NLRP3 inflammasome in nonsurgical AF are also necessary.

## Future Implications

Currently, the American College of Cardiology (ACC)/American Heart Association (AHA)/Heart and Rhythm Society (HRS) Guideline for the Management of AF gives a class IIb recommendation to treat individuals with colchicine after cardiac surgery to prevent AF.^43^^,^^44^ Following data from Yao and colleagues^13^ linking the NLRP3 inflammasome to AF, and multiple cardiovascular outcome trials demonstrating a benefit with anti-inflammatory therapies for the secondary prevention of ASCVD, further investigation is required to determine a role for anti-inflammatory therapies for prevention and treatment of AF.

Given the recent evidence from LoDoCo, COLCOT, and LoDoCo 2, colchicine could be considered for primary and secondary prevention of ASCVD.^45^ High-quality RCTs are necessary to determine the efficacy of colchicine therapy for prevention and treatment of AF before it can receive a strong recommendation in future ACC/AHA and HRS guidelines. Colchicine and other anti-inflammatory agents that target the NLRP3 inflammasome pathway may prove to be a valuable tool in reducing overall AF burden and preventing progression of AF. However, given the overlap in risk factors for ASCVD and AF, addressing the modifiable risk factors will remain the cornerstone of therapy for prevention of AF.

## Funding Sources

This research did not receive any specific grant from funding agencies in the public, commercial, or not-for-profit sectors.

## Disclosures

H.C. is a consultant for Medtronic Inc and St. Jude Medical/Abbott. H.C. receives research support from Boston Scientific Corp.

## Authorship

All authors attest they meet the current ICMJE criteria for authorship.
